# A study of skin sensitivity to various allergens by skin prick test in patients of nasobronchial allergy

**DOI:** 10.4103/0970-2113.53228

**Published:** 2009

**Authors:** R. Prasad, S. K. Verma, R. Dua, S. Kant, R.A.S Kushwaha, S. P. Agarwal

**Affiliations:** *Department of Pulmonary Medicine, K.G. Medical University, Lucknow, UP, India*; 1 *Department of ENT, K.G. Medical University, Lucknow, UP, India*

**Keywords:** Skin prick test, nasobronchial allergy, allergens

## Abstract

**Objective:**

To study skin sensitivity to various allergens in patients of nasobronchial allergy.

**Materials and Methods:**

2880 skin prick tests with 60 allergens were performed in 48 patients of nasobronchial allergy.

**Results:**

Most common offending allergens were insects (21.2%), followed by dusts (12.0%), pollens (7.8%), animal dander (3.1%), and fungi (1.3%). The common insect antigen were locust female (33.3%) followed by locust male (25%), grasshopper (20.8%), cricket (16.7%), cockroach female (16.7%) and cockroach male (14.6%). Common dust allergens were house dust, wheat dust, cotton mill and paper dust. Among pollens, *Amaranthus spinosus, Argemone mexicana, Adhatoda vasica, Ailanthus* and *Cannabis* were found to be common allergens. In animal danders common offending allergens were cow dander and dog dander. Among fungi *Aspergillus fumigatus, Aspergillus flavus, Alternaria teneis* and *Fusarium sodani* were common allergens. Patients of bronchial asthma had associated allergic rhinitis in 80% cases.

**Conclusion:**

Common allergens in patients of nasobronchial allergy were identified. The data may prove useful in of allergen avoidance and immunotherapy in these patients.

## INTRODUCTION

Over 20% of the world population suffers from immunoglobulin E (IgE) mediated allergic diseases such as asthma, rhino conjunctivitis, eczema and anaphylaxis.[1] In India alone, approximately 20% of the population suffers from allergic rhinitis and 15% from bronchial asthma. Airway allergy is now considered to be a disease not confined to a specific target organ but rather a disorder of the whole respiratory tract. Epidemiological evidences and clinical as well as experimental observations have suggested a link between rhinitis and asthma leading to a definition of allergic rhinobronchitis[2] or united airways diseases (UAD)[3] and the concept of ‘one airway one disease’.

The prevalence of nasobronchial allergy has increased in last two to three decades possibly due to change in indoor and outdoor environment. Allergens are one of the many factors, which can cause and trigger nasobronchial allergy. Prick may be very useful to identify the offending allergen. The present study was done to study the skin sensitivity to various allergens by skin prick test in patients of nasobronchial allergy.

## MATERIALS AND METHODS

### Subjects

The study was conducted among 48 patients suspected to have nasobronchial allergy and attending the Department of Pulmonary Medicine, King George's Medical University, Lucknow, from August 2004 to September 2005. Pregnant and lactating females were excluded from the study. All the patients were subjected to detailed history and clinical examination, TLC, DLC, total serum IgE, stool examination, chest X-ray PA view, spirometry (pre and postbronchodilator) and detailed ENT examination including X-ray of paranasal sinuses.

### Skin sensitivity tests

The antigens were obtained from All Cure Pharma Pvt. Ltd., New Delhi. The antigens included 32 types of pollens, 14 types of fungi, 6 types of insects, 4 types of dusts, and 4 types of dander. Oral antihistaminic were stopped two days prior and oral sympathomimetic were stopped for at least 12 hours before skin prick test. If a patient was on oral steroids continuously for more than two weeks, the skin prick test was performed three weeks after the steroid therapy had been stopped.

Drop of each allergen was placed 2cm apart and then pricked with 26 gauge needle. Buffer saline was used as a negative control while histamine acid phosphate (1 mg/ml) as a positive control. Grading of skin prick test reaction was done by comparison to histamine positive control, as grade + means 25% of area of wheal induced by histamine, + + means 50% of area of wheal induced by histamine, + + + means 100% of area of wheal induced by histamine, + + + + means 200% of area of wheal induced by histamine.[3] Only 2+, 3+, and 4+ reactions were labeled as positive skin reactions, because of high incidence of 1+ in the nonallergic normal persons also. The positive skin reactions which corelated well with the history were considered as clinically significant reactions.[4]

## RESULTS

Out of 48 patients, 28 were males and 20 were females. All the patients were between 12 to 45 years of age and majority (56%) were less than 30 years of age. 80% patients of bronchial asthma had associated allergic rhinitis, while 25% patients of allergic rhinitis had associated bronchial asthma.

Out of 48 patients 5 patients gave negative skin prick test to all the antigens tested while 43 patients various grades of positive reaction to one or more allergen. In the present study, a total of 2,880 tests were performed with 60 allergens (pollens, fungi, insects, dusts, and danders), on 48 patients of nasobronchial allergy. The results of skin reactivity are shown in Table 1.

**Table 1:** Results of skin prick tests to various allergens

Insects	Total tests	Total positives	% positive	1+	2+	3+	4+	Marked positive (2+ to 4+)	% marked positive
Cockroach (female)	48	10	20.8	2	8	0	0	8	16.66
Cockroach (male)	48	8	16.66	1	7	0	0	7	14.58
Cricket	48	10	20.8	2	7	1	0	8	16.66
Grasshopper	48	10	20.8	0	10	0	0	10	20.8
Locust (female)	48	17	35.41	1	14	2	0	16	33.33
Locust (male)	48	13	17.08	1	12	0	0	12	25
Total insects	288	68	23.61	7	58	3	0	61	21.18
Dust									
Cotton mill dust	48	4	8.33	1	1	2	0	3	6.25
Grain dust (wheat)	48	7	14.58	1	4	0	2	6	12.5
House dust	48	12	25	0	10	2	0	12	25
Paper dust	48	4	8.33	2	2	0	0	2	4.16
Total dust	192	27	14.06	4	17	4	2	23	11.97
Pollen									
*Adhatoda vasica*	48	9	18.75	0	9	0	0	9	18.75
*Ailanthus excelsa*	48	10	20.83	4	6	0	0	6	12.5
*Amaranthus spinosus*	48	19	39.58	2	15	0	2	17	35.41
*Argemone Mexicana*	48	13	27.08	2	10	0	1	11	22.91
*Azadirecta indica*	48	3	6.25	2	1	0	0	1	2.08
*Brassica campestris*	48	7	8.75	6	1	0	0	1	2.08
*Cannabis sativa*	48	7	8.75	3	4	0	0	4	8.33
*Cenchrus ciliaris*	48	10	20.83	4	5	0	1	6	12.5
*Chenopodium murale*	48	6	12.5	3	2	0	1	3	6.25
*Chenopodium album*	48	5	10.41	1	3	0	1	4	8.33
*Cynodon dactylon*	48	6	12.5	2	4	0	0	4	8.33
*Cyperus rotundus*	48	6	12.5	3	3	0	0	3	6.25
*Dodanaea viscosa*	48	7	8.75	2	5	0	0	5	10.41
*Eucalyptus tereticornis*	48	3	6.25	0	3	0	0	3	0
*Gynandropsis gynandra*	48	1	2.08	1	0	0	0	0	0
*Imperata cylindrical*	48	4	8.33	2	2	0	0	2	4.16
*Lawsonia enermis*	48	3	6.25	1	2	0	0	2	4.16
*Melia azedarach*	48	4	8.33	1	3	0	0	3	6.25
*Morus alba*	48	3	6.25	1	1	1	0	2	4.16
*Parthenium hysterophorus*	48	2	4.16	0	2	0	0	2	4.16
*Prosopis juliflora*	48	6	12.5	0	6	0	0	6	12.5
*Putranjiva roxburghii*	48	5	10.41	3	2	0	0	2	4.16
*Ricinus communis*	48	3	6.25	0	3	0	0	3	6.25
*Rumex dentatus*	48	2	4.16	0	2	0	0	2	4.16
*Sorghum vulgare*	48	4	8.33	1	3	0	0	3	6.25
*Typha angustata*	48	0	0	0	0	0	0	0	0
*xanthium srumarium*	48	7	14.58	3	4	0	0	4	8.33
*Zea mays*	48	3	6.25	1	2	0	0	2	4.16
*Ageratum conyzoides*	48	2	4.16	0	2	0	0	2	4.16
*Cassia siamea*	48	5	10.41	3	2	0	0	2	4.16
*Pennisetum typhoides*	48	3	6.25	1	2	0	0	2	4.16
*Holoptelea integrifolia*	48	7	14.58	2	4	0	1	5	10.41
Total pollen	1536	175	11.39	54	113	1	7	121	7.8
Dander									
Buffalo dander	48	1	2.08	0	1	0	0	1	2.08
Cow dander	48	3	6.25	1	2	0	0	2	4.16
Dog dander	48	3	6.25	1	2	0	0	2	4.16
Horse dander	48	1	2.08	0	0	0	1	1	2.08
Total dander	192	8	4.16	2	5	0	1	6	3.12
Fungi									
*Alternaria teneis*	48	3	6.25	1	2	0	0	2	4.16
*Aspergillus flavus*	48	3	6.25	3	0	0	0	0	0
*Aspergillus fumigates*	48	4	8.33	2	1	0	1	2	4.16
*Aspergillus niger*	48	2	4.16	0	2	0	0	2	4.16
*Candida albicans*	48	1	2.08	1	0	0	0	0	0
*Cladosporium herbarum*	48	0	0	0	0	0	0	0	0
*Curvularia lunata*	48	2	4.16	1	1	0	0	1	2.08
*Fusarium solanii*	48	3	6.25	1	2	0	0	2	4.16
*Helminthosporium*	48	0	0	0	0	0	0	0	0
*Mucor mucedo*	48	0	0	0	0	0	0	0	0
*Neurospora sitophila*	48	2	4.16	2	0	0	0	0	0
*Pencillium species*	48	0	0	0	0	0	0	0	0
*Rhizopus nigricans*	48	1	2.08	0	1	0	0	1	2.08
*Trichoderma species*	48	2	4.16	2	0	0	0	0	0
Total fungi	672	23	3.42	13	9	0	1	10	1.33

## DISCUSSION

Out of 48 patients of nasobronchial allergy, in whom skin prick test was performed, 10.4% of the patients showed negative reaction to all antigens tested, while the remaining 89.6 showed positive reactions of various grades. Markedly, the positive skin reactions (2+ to 4+) were quite common for the various allergens tested. The common offending allergens found in the study were insects (21.8%), followed by dusts (11.9%), pollens (7.8%), dander (3.1%), and fungi (1.3%) [Table 1]. In a previous study done at the same centre, insects (17.5%), dusts (15.4%), danders (13.8%), pollens (10.9%), and fungi (10.3%) were found to be common offending allergens among patients of bronchial asthma by intradermal skin tests.[5] Holopainen *et al.,*[6] studied the distribution of allergens in patients of seasonal and perennial allergic rhinitis. Sensitivity to house dust was present in 44%, pollens in 30-40%, mite extract in 10%, and moulds in 9%. Animal danders were not found to be of great importance. Hendricks *et al.*,[7] analyzed skin prick test reactions in 656 asthmatic patients. Positive skin prick tests were reported to pollens in 66%, animal danders in 38%, Aspergillus fumigatus in 16%, and other moulds in 21%. There was a highly significant association of positive history with positive prick test for all allergens studied. The variation in the results can be very well understood with the fact that there is topographical variation.

In the present study, the markedly positive skin reaction to pollen antigens varied from 0 to 35.4% (average 7.8%) in patients of nasobronchial allergy. The most common among pollens was Amaranthus spinosus (35.4%), followed by Argemone mexicana (22.9%), Adhatoda vasica (18.5%), Ailanthus (12.5%), and Cannabis (8.3%). Gynandropsis gynandra, Eucalyptus, and Typha angusta did not show any markedly positive skin reaction indicating that they are not the common pollen allergens in this part of country. A similar study was done by Agnihotri *et al*.,[8] in Lucknow during 1969-1971 to know the allergenicity of various pollen in allergic patients. Comparison with this study revealed that positive skin reactions to pollens such as Amaranthus were common in both studies. In the present study, some new pollens (Argemone, Adhatoda, Ailanthus, and Cannabis) were found showing markedly positive reaction. Singh *et al*.,[9] studied the common environmental allergens causing respiratory allergy in atmospheric surveys and found that Alanus nitida, Amaranthus spinosus, Argemone mexicana, Cocos, Carica, Cedrus, Cassia, Parthenium, Chenopodium album, Dodonaea, Prosopis julifora, Ricinus communis, and Holoptelia integrifolia are the important pollens. Shivpuri[10] reported that the pollens, Holoptelea, Sorghum vulgare, Pennisetum, Artemisia, Ricinus communis, Morus alba, Cassia, Argemone mexicana, Cyanodon dactylon, Chenopodium, Brassica campestris, Cassia, Cenchrus, Carica, Cannabis, Xanthium strumarium, Amaranthus, Imperata, and Putranjiva are the common pollens in the patients of nasobronchial allergy.

Among fungi, markedly positive skin reaction varied from 0-4.6% (average 1.3%), and the common fungal antigens were Aspergillus fumigates, followed by Aspergillus flavus, Alternaria teneis, and Fusarium sodani. Also, Aspergillus niger, Candida albicans, Cladosporium herbarum, Helminthosporium, Mucor mucedo, Neurospora, Pencillium, and Tricoderma did not show markedly positive reaction in any patients of nasobronchial allergy. Singh *et al*.,[11] conducted a study in Lucknow in 1980 to know the common fungal spores prevalent in the city and to find out their allergenicity in allergic patients. Comparision with this study revealed that allergens such as Fusarium and Aspergillus were common in both studies. In our study, some new fungal allergens such as Alternaria showed markedly positive reaction. Shivpuri *et al*.,[4] found Curvalaria spp, Alternaria, Aspergillus fumigatus, Phoma, Neurosporaspp, Aspergillus tamari, Helminthosporum, Aspergillus niger, Rhizopus nigricans, Trichoderma, and Cladosporium, to be the common fungal allergen in patients of nasobronchial allergy. Agashe SN[12] found mold spores of Cladosporium, Periconium, Nigrospora, Alternaria, Helminthosporium, Smut spores, Aspergillus, and Penicillium to be the common aeroallergens.

High rates of markedly positive skin reaction were also shown by insects (21.2%), female locust (33.3%), male locust (25%), grasshopper (20.8%), cricket (16.7%), female cockroach (16.7%), and male cockroach (14.6%). Gaur *et al.,*[13] found allergy to moth, mosquito, locust (male), locust (female), dragonfly, jassids, housefly, cockroach grasshopper, wasp, beetle, ant, cricket, honeybee to be common in patients of nasobronchial allergy. Achary[14] also found moth, cockroach, mosquito, and ant to be common insect allergens among patients of nasobronchial allergy. Only 3.12% of patients with nasobronchial allergy showed markedly positive skin test to various animal danders in the present study, while it was reported in 13.8% in previous study done at same centre using intradermal skin test.

In the present study, 12% patients of nasobronchial allergy showed markedly positive skin reaction to various dusts. Most common dusts were house dust (25%), followed by wheat dust (12.5%), cotton dust (6.3%), and paper dust (4.2%). Duc *et al.*,[15] found total house dust to be the most common allergen in patients of rhinitis with bronchial asthma followed by grass pollens, HDM, and animal dander. Acharya *et al.,*[14] found house dust followed by wheat dust, cotton dust and paper dust to be common among patients of nasobronchial allergy.

The differences in incidence of markedly positive reactions in various studies may be due to different flora in different geographical areas and change of flora over a successive time period due to change in the climatic factors. The present study and such studies will definitely be helpful in identifying the allergens causing various allergic disorders including nasobronchial allergy in this part of the country. Furthermore, this study may also be useful in avoidance of allergens causing allergy and allergen immunotherapy. Further studies may be continued from time to time in this part of country to know the newer allergens causing allergic disorders.
